# Functional Hydrogels With Tunable Structures and Properties for Tissue Engineering Applications

**DOI:** 10.3389/fchem.2018.00499

**Published:** 2018-10-22

**Authors:** Xiaomeng Li, Qingqing Sun, Qian Li, Naoki Kawazoe, Guoping Chen

**Affiliations:** ^1^School of Mechanics and Engineering Science, Zhengzhou University, Zhengzhou, China; ^2^National Center for International Joint Research of Micro-nano Moulding Technology, Zhengzhou University, Zhengzhou, China; ^3^Center for Functional Sensor and Actuator, National Institute for Materials Science, Tsukuba, Japan; ^4^Research Center for Functional Materials, National Institute for Materials Science, Tsukuba, Japan

**Keywords:** functional hydrogels, tissue engineering, physical properties, chemical properties, microstructures

## Abstract

Tissue engineering (TE) has been used as an attractive and efficient process to restore the original tissue structures and functions through the combination of biodegradable scaffolds, seeded cells, and biological factors. As a unique type of scaffolds, hydrogels have been frequently used for TE because of their similar 3D structures to the native extracellular matrix (ECM), as well as their tunable biochemical and biophysical properties to control cell functions such as cell adhesion, migration, proliferation, and differentiation. Various types of hydrogels have been prepared from naturally derived biomaterials, synthetic polymers, or their combination, showing their promise in TE. This review summarizes the very recent progress of hydrogels used for TE applications. The strategies for tuning biophysical and biochemical properties, and structures of hydrogels are first introduced. Their influences on cell functions and promotive effects on tissue regeneration are then highlighted.

## Introduction

Tissue engineering (TE) has emerged as a useful approach to treat tissue damages caused by diseases and trauma, which has shown many advantages as compared to conventional treatment strategies. To afford desirable therapeutic outcome, scaffolds prepared from various kinds of biomaterials have been used for TE to accommodate sufficient amount of cells and to control cell functions (Chen and Kawazoe, 2016a,b; Chen et al., 2018).

Among of different types of scaffolds, hydrogels have attracted more and more attention in the TE field owing to their similarity to *in vivo* cellular microenvironment and tunable physiochemical properties (Drury and Mooney, 2003). Hydrogels are generally prepared by translating hydrophilic polymers solution into 3D network structure via physical or chemical crosslinking. During this process, hydrogels can encapsulate cells homogeneously and provide cells a 3D microenvironment similar to the native extracellular matrix (ECM) (Tibbitt and Anseth, 2009). Cell behaviors and functions *in vivo* are affected by the stimuli that are produced by the surrounding ECM. In a similar way, the structures and physiochemical properties of hydrogels provide critical cues to control the functions of embedded cells and thus guide tissue regeneration.

The structures and physiochemical properties of hydrogels can be designed and controlled through selecting different biomaterials, crosslinking methods and fabrication strategies. This review summarizes the latest developments of functional hydrogels for TE applications. At first, the materials and crosslinking methods used for hydrogel preparation are introduced. And then, the approaches to tune the structure and physiochemical properties of hydrogels and their effects on cell functions and tissue regeneration are compared. Finally, the challenges and future perspectives of functional hydrogels are discussed.

## Materials and crosslinking methods for functional hydrogels preparation

There are several criterias to prepare hydrogels for TE applications. Firstly, the materials and crosslinking agents should be compatible toward living cells and biological factors (e.g., growth factors). Secondly, the preparation process should be easily occured under mild conditions. Thirdly, the products from hydrogel degradation should be non-toxic to cells and tissues. The materials and crosslinking methods used for hydrogel preparation for TE are summarized in Table 1.

**Table 1:** Materials and crosslinking methods for functional hydrogels preparation.

**Materials**			**Crosslinking methods**		**TE applications**	**References**
		**Characteristics**	**Physical crosslinking**	**Chemical crosslinking**		
Natural Materials	Gelatin		Guest-host		Cartilage TE	Feng et al., 2016
		GelMA		Photo	Cartilage, tendon TE	Yang et al., 2016; Li et al., 2017b
		GelMA	Thermal	Photo	Corneal TE	Rizwan et al., 2017
		Gelatin-hydroxyphenylpropionic acid (Gtn-HPA)		Enzymatic	Cartilage TE	Wang et al., 2014; Le Thi et al., 2017
	Collagen			Photo	Meniscus TE	Heo et al., 2016
	HA	Adamantane-functionalized HA	Guest-host		Cartilage TE	Wei et al., 2016
		HA-vinyl sulfone		Michael addition	Neural engineering	Shendi et al., 2016
		Maleimide-functionalized HA and furan-functionalized HA		Diels-Alder	Adipose TE	Fan et al., 2015a
		Methacrylated HA (MeHA)		Photo	Meniscus, nucleus pulposus TE	Kim et al., 2015; Koh et al., 2017
	Chondroitin sulfate	Furfurylamine grafted chondroitin sulfate		Diels-Alder	Bone TE	Bai et al., 2017
		Methacrylated chondroitin sulfate		Photo	Cartilage TE	Levett et al., 2014; Abbadessa et al., 2016
	Alginate		Ionic		Cartilage, retinal and bone TE	Chaudhuri et al., 2016; Hunt et al., 2017; Lee et al., 2017
		Methacrylated alginate		Photo	Bone TE	Ho et al., 2016
	Dextran	Dextran bifunctionalized with methacrylate and aldehyde		Photo	Vascular TE	Liu and Chan-Park, 2009
		Azadibenzocyclooctyne-modified dextran and azide-modified dextran		Alkyne-azide	Cartilage TE	Wang et al., 2017b
	Agarose		Thermal		Osteochondral, skin TE	Sheehy et al., 2013; Miguel et al., 2014
	Chitosan	Chitosan-g-poly(N-isopropylacrylamide)	Thermal		Cardiac, cartilage TE	Baei et al., 2016; Mellati et al., 2017
		Methacrylated glycol chitosan		Photo	Bone TE	Kim et al., 2016
Synthetic and Hybrid Materials	PEG	Norbornene-terminated PEG		Michael addition	Cartilage, vascular TE	Mahadevaiah et al., 2015; Sridhar et al., 2015; Wang et al., 2017a
		Thiol-norbornene PEG, PEG diacrylate (PEGDA)		Photo	Cartilage TE Heart valve TE	Zhang et al., 2015; Neumann et al., 2016
	PAM/Gelatin	PAM and GelMA		Photo	Cartilage TE	Han et al., 2017
	PVA/Gelatin		Thermal		Cartilage TE	Kim et al., 2016
	PNIPAM/Gelatin	PNIPAAm-based copolymer, thiol-modified gelatin	Thermal	Michael addition	Cardiac TE	Navaei et al., 2016b
	PEG/Gelatin	PEG dimethacrylate, GelMA		Photo	Bone, cartilage TE	Gao et al., 2015
	PEG/Chitosan	Glycol chitosan, benzaldehyde functioned PEG		Schiff-base	Neural engineering, vascular TE	Tseng et al., 2015; Hsieh et al., 2017
	PEG/HA	Amino-terminated PEG, aldehyde HA		Schiff-base	Adipose TE	Fan et al., 2015b
		Furylamine and tyramine functional HA; Dimaleimide PEG		Diels-Alder/ Enzymatic	Cartilage TE	Mahadevaiah et al., 2015
	Gelatin/HA	GelMA/MeHA		Photo	Skin, neural TE	Eke et al., 2017; Magarinos et al., 2018
	Gelatin/Alginate	Oxidized alginate, gelatin		Schiff-base	Muscle TE	Baniasadi et al., 2016
		GelMA and alginate		Photo	Bone TE	Lewandowska-Łancucka et al., 2017
		GelMA, alginate	Ionic	Photo	Bone TE	Pacelli et al., 2018

### Hydrogel construction materials

Materials used to prepare TE hydrogels can be briefly classified into natural and synthetic polymers. Hydrogels prepared from natural polymers possess intrinsic advantages, such as high biocompatibility, biodegradability and similar microenvironment to that of native tissues. Natural polymers used for hydrogel preparation include protein-based materials (such as gelatin, collagen, fibrin, and silk fibroin) and polysaccharide-based materials (such as hyaluronic acid (HA), chondroitin sulfate (CS), alginate, chitosan and so on). Collagen, as the main ECM component of various tissue, is an attractive material for hydrogels preparation (Heo et al., 2016). It's derivative, gelatin, is also a frequently used protein-based material for hydrogel formation, which has higher solubility and lower cost when compared with collagen (Zhao et al., 2016). Gelatin-based hydrogels are good candidates for various TE. For example, injectable gelatin methacryloyl (GelMA) hydrogels are prepared for cartilage TE (Li et al., 2016). The chondrocytes 3D cultured in these hydrogels have shown excellent viabilities and desirable functions. HA as a glycosaminoglycan is commonly prevalent in body liquid and native ECM (Shendi et al., 2016). Therefore, it has been used to prepare various kinds of hydrogels for cartilage, skin, and many other TE. For example, it has been reported that HA molecular can bond with mesenchymal stem cells (MSCs) through CD44 receptors and promote the chondrogenesis (Chung and Burdick, 2008). CS is a sulfated glycosaminoglycan with a linear structure existing in cartilage tissue ECM (Levett et al., 2014). Chondrocytes cultured in CS hydrogels have round morphology, enhanced gene expression, and secretion of cartilaginous ECM (Levett et al., 2014; Zhu et al., 2014). Other polysaccharide-based materials, such as alginate (obtaining from bacteria and brown seaweed) and chitosan (derived from chitin that is produced from the shells of crabs and shrimps), are also commonly used for hydrogel preparation due to their biocompatibility, degradability and easy modification (Kim et al., 2016; Hunt et al., 2017).

On the other hand, synthetic polymers have also exhibited wide usage due to their controllability, reproducibility, and good mechanical properties (Guan et al., 2017). Representative synthetic polymers used for TE hydrogels include poly(ethylene glycol) (PEG), poly(vinyl alcohol) (PVA),poly (N-isopropylacrylamide) (PNIPAM) (Haq et al., 2017), and polyacrylamide (PAM) (Darnell et al., 2013). PEG and PVA have low toxicity, making them widely used for cell-laden hydrogels and drug carriers (Kim et al., 2016; You et al., 2018). However, due to the lack of biological activity, the biocompatibility of these synthetic polymers is compromised relative to natural materials. Hybridization with natural materials is an efficient and easy-going approach to integrate the advantages of natural and synthetic component. It has been reported that the biocompatibilities of PAM, PVA, PNIPAM, and PEG hydrogels are significantly improved after blend with gelatin (Gao et al., 2015; Kim et al., 2016; Navaei et al., 2016b; Han et al., 2017). The cell spreading, and proliferation in these hybrid hydrogels are enhanced compared with the cells 3D cultured in synthetic hydrogels. Besides, the hybridization of different natural materials can also exhibit novel attractive properties. For example, alginate is interpenetrated and crosslinked into photopolymerized gelatin hydrogel network to generate alginate/gelatin hybrid hydrogel (Pacelli et al., 2018). Such a hydrogel is confirmed to have the function of promoting bone tissue regeneration due to the good biocompatibility and enhanced mechanical property.

### Crosslinking methods

There are various methods for crosslinking of hydrophilic polymers chains to form hydrogels, which usually are selected depending on the materials chemistry and expected functions. In general, they can be classified into physical and chemical crosslinking methods.

#### Physical crosslinking methods

Physically crosslinked hydrogels can be prepared at very mild conditions without the utilization of crosslinking agents that often cause toxicity to cells or may affect the activity of biological molecules encapsulated in hydrogels (Hennink and Van Nostrum, 2012). There are many methods to prepare physically crosslinked hydrogels for TE applications, such as ionic interaction, guest-host interaction and thermo-gelation.

##### Ionic crosslinking

Ionic crosslinking method is frequently used to encapsulate cells and drugs due to the mild crosslinking procedures (Goosen et al., 1985; Gombotz and Wee, 2012). The most representative hydrogels formed by ionic crosslinking is alginate hydrogel. The crosslinking is through the exchange of sodium ions from guluronic acid units with divalent cations such as calcium to form junction zones (Gacesa, 1988). This physically crosslinked alginate hydrogel not only has shown a biocompatible property but also has many other functions, such as self-healing and stress relaxation, due to the reversible dissociation and re-bonding between α-L-guluronic acid in alginate and calcium ions. However, the stability of ionic hydrogel should be considered when using under physiological conditions. For instance, calcium crosslinked alginate hydrogel will lose stability in 0.9 wt% sodium chloride solution due to the exchange of calcium ions by sodium ions (Martinsen et al., 1989).

##### Guest-host chemistry

The guest-host chemistry holds great potential for hydrogels preparation, because the low cytotoxicity and specific selectivity. In this reaction, host molecules can selectively recognize and physically bind to certain guest molecules to form crosslinked network. The interaction includes hydrophobic association, hydrogen bonding, electrostatic interaction, van der Waals forces, and so on (Steed et al., 2007). Cyclodextrin, as one macrocycle, has hydrophobic interior cavities that have a high affinity for specific hydrophobic guest moieties. Specifically, guest–host pair, adamantane (guest) and β-cyclodextrin (host), is widely used to synthesize corresponding macromers. The guest-host bonding can translate mixed solution into hydrogels easily (Rodell et al., 2016). In another study, acrylate β-cyclodextrin can build guest-host binding with the benzene ring of gelatin to form supramolecular hydrogels. These hydrogels have very good bio-adhesive property and sustainable release of hydrophobic drugs, which is promising for TE of bone, cartilage and tendon (Feng et al., 2016).

##### Thermo-gelation

Thermo-gelation is to build a physically crosslinked network by altering the temperature. For example, the crystalline nature of PVA has been applied to fabricate physically crosslinked hydrogel by several repeating cycles of freezing and thawing (Hassan and Peppas, 2000). These physically crosslinked PVA hydrogels are reported to have many characteristics, such as high swelling degree and mechanical strength. Gelatin solution becomes hydrogel due to the intermolecular hydrogen bonding formation when the temperature drops below the upper critical solution temperature (UCST). In contrast, some other macromer, such as PNIPAM, becomes hydrogel when temperature increases above the lower critical solution temperature (LCST) due to the balance between hydrogen bonding and hydrophobic effects (Ashraf et al., 2016). These temperature-responsive polymers can transform phases between sol and gel near physiological temperature, which enables the hydrogels injectable. PNIPAM-based injectable hydrogel has been used to encapsulate cardiomyocytes and the encapsulated cells exhibit high viability and mature phenotypes (Navaei et al., 2016b).

#### Chemical crosslinking methods

Chemically crosslinked hydrogels have a better performance at stability than physically crosslinked hydrogels due to stronger binding energy and substantially improved flexibility. Hydrogel-forming water-soluble polymers have many functional groups, such as OH, COOH, and NH_2_. 3D network can be established by covalent bonding between these functional groups using glutaraldehyde and EDC/NHS (Balakrishnan et al., 2013; Omobono et al., 2015; Cheaburu Yilmaz et al., 2017). However, the toxicity of crosslinking agents and process limit the TE applications. For example, small molecular crosslinkers like glutaraldehyde and carbodiimides have been reported to be toxic and are not recommended to fabricate cell-laden hydrogels (Balakrishnan, 2016). To meet the requirements for TE, several available strategies for functional hydrogel preparation will be discussed below.

##### Photopolymerization

Photopolymerization has been widely used for hydrogel fabrications due to its biocompatibility and spatiotemporal controllability. Usually, macromers are modified with photoreactive moieties, such as methacrylate or acrylate groups. The photoreactive macromer solution with photoinitiator can be crosslinked under UV or visible light. The photoinitiators can generate free radicals that are transferred to the photoreactive carbon double bond groups in the modified macromers to start chain polymerization. However, under high photo exposure, photoinitiator will generate a large number of free radicals which may react with intracellular molecules to induce cell damage. This problem can be addressed by decreasing the light energy and amount of photoinitiator (Bryant et al., 2000; Fedorovich et al., 2009). High density of methacrylate groups has been reported to protect encapsulated cells by quenching free radicals (Bartnikowski et al., 2015). The method can be used for the preparation of hydrogels from various kinds of polymers and a variety of cell types can be encapsulated into the hydrogels for TE applications (Park et al., 2003; Chen et al., 2018). One of the most frequently used macromers is GelMA, which retains cell adhesive peptide sequence (arginine-glycine-aspartic acid, RGD) and matrix metalloproteinase (MMP) degradable peptide (Yue et al., 2015).

##### Enzyme-enabled crosslinking

Enzyme-enabled crosslinking is highly selective for a specific enzyme and can be achieved under mild physiological conditions (Ulijn, 2006). Transglutaminase and horse radish peroxidase are two commonly used enzymes. Transglutaminase catalyzes trans-amidation reaction that introduces N ϵ-(γ- glutamyl)lysine cross-links in proteins, converting protein solution into 3D hydrogel network (Chen et al., 2003). Horse radish peroxidase can build networks between polymers by oxidative coupling of hydroxyphenylpropionic acid moieties (Wang et al., 2014). Besides selectivity, this enzyme-mediated crosslinking approach also exhibits rapid gelation and easily tunable mechanical properties by varying the concentration of horse radish peroxidase and H_2_O_2_ (Ren et al., 2015).

##### Click chemistry

Click chemistry has the characters of high efficiency, high yield, and proceeding with no side products, which make the method wildly studied. TE application requires the reaction to proceed under physiological conditions. The typical click reaction is thiol-ene radical reaction which is hindered by inhibitory capacity of oxygen and shows complicated volume relaxation and stress development compared with classical radical photopolymerization (Hoyle and Bowman, 2010). Other representative chick chemistry includes Diels-Alder reaction, azide-alkyne cycloaddition chemistry (Xu and Bratlie, 2018) and Michal addition. Strain-promoted azide-alkyne cycloaddition (SPAAC) click chemistry has drawn increasing attention recently due to the mild reaction conditions and bioorthogonality (Xu et al., 2014). An injectable and degradable PEG-based hydrogel has been prepared via the bioorthogonal SPAAC click chemistry (Jiang et al., 2015). Michael addition reaction is the nucleophilic addition of a carbanion or a nucleophile to an α, β-unsaturated carbonyl compound. Michael addition-mediated hydrogels can be prepared under physiological conditions, making this kind of hydrogels injectable (Sun et al., 2017). For example, HA with thiol functional groups can form a 3D network with PEG vinysulfone macromers via Michael addition. The gelation time can be controlled by the degree of functionalization and the ratio of the two functional groups (Jin et al., 2010).

##### Schiff base reaction

Schiff base reaction has been widely used to form hydrogels via the coupling between aldehyde and amine groups in polymer chains. These hydrogels have been reported to have self-healing capacity due to the dynamic equilibrium of the linkages. For example, glycol chitosan and benzaldehyde functioned PEG have been synthesized and used to form a hydrogel with self-healing property for central nervous regeneration (Tseng et al., 2015). Proliferation rate and differentiation tendency of neurosphere-like progenitors are enhanced in the self-healing hydrogel.

The above-described materials and crosslinking reactions have their respective merits and demerits. While, rational combination of natural and synthetic polymers or different crosslinking methods may afford an optimized approach to improve the hydrogel functions. For example, enzymatic crosslinking and Diels-Alder click chemistry can be induced into HA/PEG hydrogels for cartilage TE (Mahadevaiah et al., 2015). Both biocompatibility and mechanical property are improved by blending of HA and PEG polymers. The enzymatic crosslinking provides a suitable gelation speed for injectability, while the click reaction generates second crosslinking that renders an outstanding shape memory and anti-fatigue property. All these characters are required for cartilage TE.

## Control of physical properties

Physical properties of hydrogels, such as mechanical strength, stiffness, stress relaxation, self-healing, and degradation, can be controlled at different levels to meet the specific requirements for TE. These physical properties have obvious effects on cell functions, and thus should be investigated and reviewed. The hydrogels prepared with different physical properties, and their effects on cell functions, as well as their applications for TE are summarized in Table 2.

**Table 2:** Physical properties of hydrogels and their performance as TE scaffolds.

**Physical properties**	**Materials**	**Approaches**	**Applications and performance**	**References**
Mechanical strength	GelMA	Chitin nanofibers, Nanoparticles blending	Strain-to-failure increased 200% after chitin nanofiber assembly; stiffness of collagen-based hydrogel increased 10-fold after addition of functionalized nanoparticles.	Jaiswal et al., 2015; Hassanzadeh et al., 2016
	PAMPS/PDMAAm	Double network	High strength PAMPS/PDMAAm gel could induce spontaneous hyaline cartilage regeneration in the osteochondral defect.	Yasuda et al., 2009; Fukui et al., 2014
Stiffness	RGD modified alginate, agarose, and PEGDA	Tuning of Ca^2+^ or polymer concentration	Intermediate stiffness promoted the osteogenic differentiation of murine MSCs.	Huebsch et al., 2010
	Four-arm maleimide-functionalized PEG and four-arm thiol-functionalized PEG	By using different PEG concentration	The proliferation, self-renewal and vascular differentiation of stem cells were enhanced in lower stiffness hydrogel.	Mahadevaiah et al., 2015
	MeHA	Tuning of macromer concentration or UV exposure time	Low stiffness of HA hydrogel promoted chondrogenic differentiation of MSCs. Highly crosslinked HA hydrogel promoted hypertrophic conversion of encapsulated MSCs.	Bian et al., 2013
	Gel-HPA	Altering macromer and/or H_2_O_2_ concentration	Medium stiffness showed superior stimulus for maintaining of chondrogenic phenotype, high stiffness promoted collagen type II gene expression.	Wang et al., 2014
	GelMA	Using the same macromer concentration with different methacryloyl substitution	High stiffness environment was beneficial for maintaining of chondrogenic gene expression.	Li et al., 2016
Stress relaxation	RGD-alginate	Tuning of stress-relaxation by using alginate with different molecular weight or PEG spacer	Fast stress relaxation promoted MSC spreading and osteogenic differentiation.	Chaudhuri et al., 2016
	Alginate	Same as above	Slow relaxing environment restricted cell volume expansion, up-regulated the gene related to matrix degradation and cell death.	Lee et al., 2017
	HA, Collagen I	Dynamic crosslinking of HA-ALD and HA-BLD, combined with collagen	Fast relaxation promoted cell spreading and focal adhesion formation.	Lou et al., 2018
Self-healing	Glycol chitosan, benzaldehyde functioned PEG	Reversible Schiff-base reaction	Self-healing hydrogel could increase proliferation and neural differentiation of neural stem cells, and enhanced capillary inducing capacity of vascular endothelial cells.	Tseng et al., 2015; Hsieh et al., 2017
		Dynamic acylhydrazone bond and DA click covalent crosslinking	Increasing the viability, decreasing apoptosis of MSCs and promoting bone regeneration	Lü et al., 2017
Degradation	GelMA	Collagenase degradable photocrosslinked gelatin hydrogel	Valvular interstitial cells had more spreading morphology in collagenase treated GelMA hydrogel than untreated hydrogel.	Benton et al., 2009
	Sulfated HA	Slowing the degradation of HA hydrogel by sulfated modification	The low degradation was beneficial for chondrogenesis of MSCs.	Feng et al., 2017
	HA functionalized with both maleimide and methacrylate	Thiol-ene crosslinking via MMP degradable crosslinker and photocrosslinking	Differentiation of MSC was directed by degradation-mediated cellular traction.	Khetan et al., 2013
	PEG-derivative	Hydrogel crosslinked by PEG derivative containing nitrobenzyl ether moieties could be degradable by photo exposure.	MSC spreading was enhanced after photodegradation.	Kloxin et al., 2010
	PEG-derivative	Modification of ends of PEG with oligo (lactic acid) and acryloyl, hydrolysis of the ester bonds altered the degradation	The high degradation enhanced osteogenesis of MSCs.	Peng et al., 2018

### Mechanical strength and stiffness

Conventional hydrogels normally possess breakable characters that will decrease their stability and thus cannot be utilized for specific tissue application such as bone, cartilage and tendon. To overcome this issue, two effective strategies have been developed. One is the hybridization of hydrogels with other polymers, nanoparticles or nanofibers. For example, regenerated silk fibroin and chitin nanofiber have been used to improve the mechanical strength of GelMA hydrogels by β-sheet folding and self-assembly, respectively. The hydrogel elastic modulus increases by one-thousand folds and strain-to-failure enhances by around 200% after chitin nanofiber assembly (Hassanzadeh et al., 2016). The hydrogels also demonstrate good cell viability, promotive cell differentiation and stable vasculature formation. Collagen-based hydrogels with a 10-fold increase in stiffness have been realized after mixing with very low amount of chemical functionalized nanoparticles working as crosslinker epicenters to make collagen chains crosslinked on the surface of nanoparticles (Jaiswal et al., 2015). Due to the interaction between nanoparticles and polymer chains, the mechanical properties of hybrid hydrogels can be enhanced. The other strategy is to prepare interpenetrating polymer network (IPN) hydrogels with high resistance to wear and high fracture strength, which has gained a lot of attention recently (Dragan, 2014). Double networks (DN) are introduced in hydrogels to enhance mechanical property for cartilage TE (Gong et al., 2003; Yasuda et al., 2009; Fukui et al., 2014). The feature of DN hydrogels is the formulation of first densely crosslinked hydrogel and second loosely network. The first network serves as sacrificial bonds to disperse the stress, while the second polymer chains work as hidden length that can extend to sustain large deformation (Haque et al., 2012). Similarly, ionic crosslinked chitosan with low molecular weight is used to work as the second crosslinking component to enhance the mechanical strength of the UV initiated PAM hydrogel (Yang et al., 2018).

**Figure 1:**
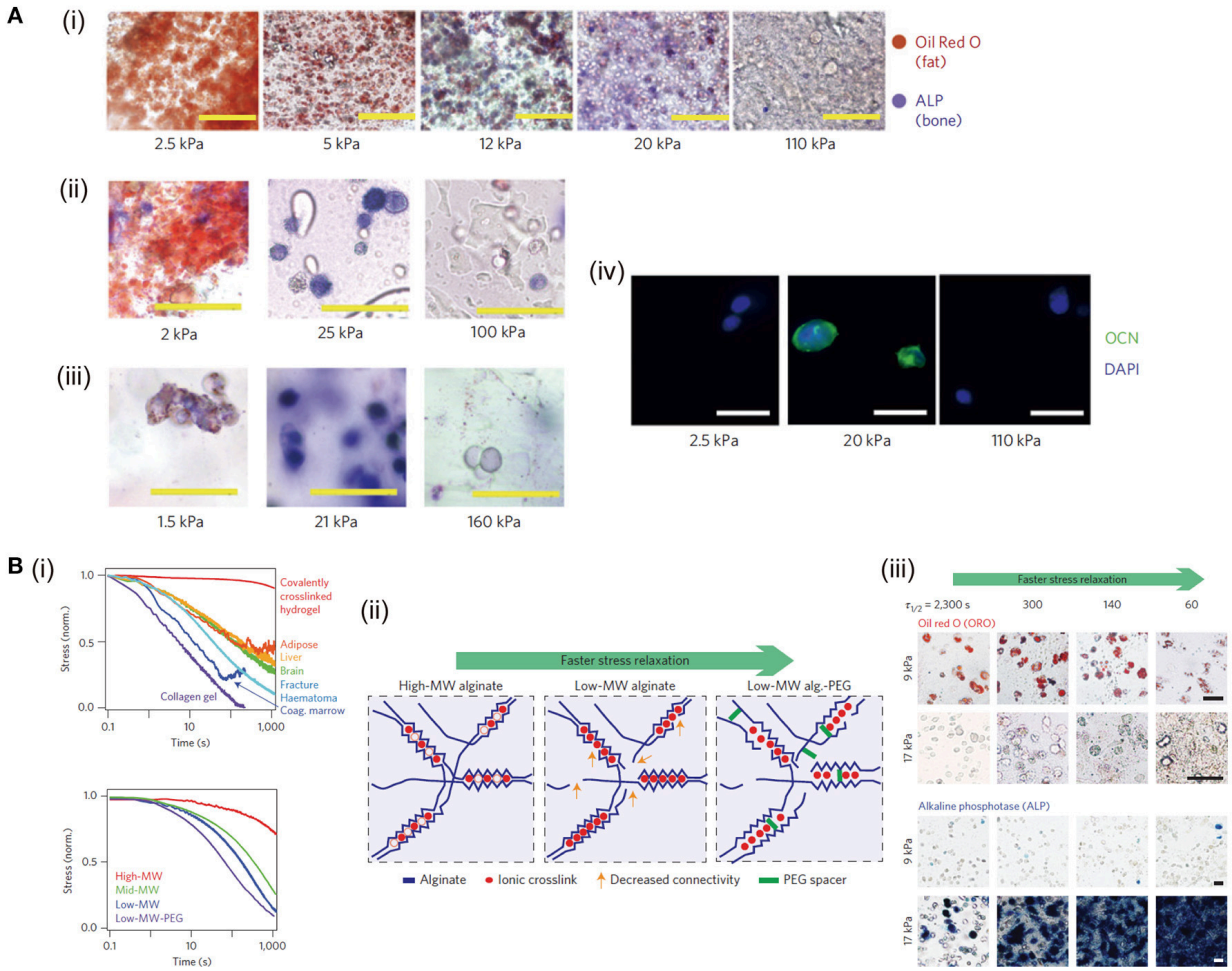
Control of stiffness and stress relaxation of hydrogels and their influence on cell functions: **(A)** MSC differentiation affected by the stiffness of RGD-modified alginate (i), RGD-modified agarose (ii), and RGD-modified PEGDA (iii) hydrogels. Alkaline phosphatase (ALP) activity (fast blue; osteogenic biomarker, blue) and neutral lipid accumulation (oil red O; adipogenic biomarker, red) staining of MSCs after 1 week of culture. Osteocalcin (OCN, green) and nuclear counterstain 4′,6-diamidino-2-phenylindole (DAPI, blue) staining in alginate hydrogel (iv). Scale bars: (i) 100 μm, (ii)–(iii) 50 μm and (iv) 20 μm, respectively. Reproduced with the permission from Huebsch et al. (2010), Copyright © 2010, Springer Nature. **(B)** Stress relaxation properties of living tissues and prepared hydrogels (i). Decreasing molecular weight (MW) of alginate and coupling PEG spacers both are predicted to increase the rate of stress relaxation (ii). MSCs cultured in hydrogels at indicated initial modulus and timescale of stress relaxation undergo adipogenic and osteogenic differentiation (Oil Red O staining and alkaline phosphatase staining) for 7 days (iii). Scale bars are 25 μm. Reproduced with the permission from Chaudhuri et al. (2016), Copyright © 2016, Springer Nature.

Macroscopically, the mechanical property is important to maintain the scaffold stability to bear loads and fulfill the defects. Meanwhile, at microscopic level, the mechanical signals play a critical role in affecting cell activities and fates. For example, matrix stiffness has been reported to affect cell spreading, migration, proliferation and differentiation (Wen et al., 2014). Stiffness of hydrogels can be tuned by changing the crosslinking density, crosslinker length and molecular weight of the precursors (Slaughter et al., 2009; Li et al., 2016, 2017b). Differentiation of mesenchymal stem cells (MSCs) cultured on 2D matrix surface depends on substrate stiffness (Engler et al., 2006). MSCs cultured in hydrogels with stiffness of lower (0.1–1 kPa), intermediate (8–17 kPa) or higher ranges (34 kPa) can differentiate into neural, myogenic or osteogenic phenotypes, respectively. However, the microenvironment provided to the cells in 3D culture differs from that in the traditional 2D system (Baker and Chen, 2012). Osteogenic differentiation of murine MSCs is enhanced in RGD modified alginate, agarose and PEG diacrylate (PEGDA) with intermediate stiffness (11–30 kPa) (Huebsch et al., 2010) (Figure 1A). Influence of stiffness on chondrogenic, vascular and neural differentiation has also been studied (Banerjee et al., 2009; Bian et al., 2013; Mahadevaiah et al., 2015). For instance, to investigate the influence of stiffness on the maintaining of chondrocyte phenotype, GelMA hydrogels with different stiffness but the same RGD density are prepared by changing the degree of methacryloyl substitution while using the same GelMA concentration. Bovine articular chondrocytes are encapsulated and cultured in the GelMA hydrogel with low, medium and high stiffness. The chondrocytes encapsulated in high stiffness hydrogel exhibit round cell morphology and high chondrogenic gene expression, while chondrocytes cultured in low stiffness counterparts show elongated morphology and low chondrogenic gene expression (Li et al., 2016).

### Stress relaxation

Stress relaxation is another important mechanical property for hydrogels, which also is a common behavior of tissue matrix. It has been reported that ionic and covalent hydrogels both exhibit stress relaxation through uncoupling-reforming of crosslinks and migration of water, respectively (Zhao et al., 2010). Ionic hydrogels have faster stress relaxation and easier modulation than that of covalent hydrogels. Sodium alginate has been commonly used to form hydrogels with tunable stress relaxation. In a previous study, RGD modified alginate has been used to prepare soft but highly stress-relaxing substrate. Cells cultured in this substrate spread similarly to the cell on the surface of stiff elastic substrates (Chaudhuri et al., 2015). The stress relaxation of covalently crosslinked PAM hydrogel, physically crosslinked collagen hydrogel and living tissues like adipose, liver, brain and an initial fracture haematoma are compared by measuring the stress change when fixing the strain at 15% (Chaudhuri et al., 2016). The results indicate that the stress relaxation of collagen hydrogel is faster than that of living tissues and PAM hydrogel, while that of PAM is slowest. RGD coupled alginate with different molecular weights and PEG spacers are used to prepare hydrogels with different stress relaxation properties independent of initial elastic modulus and matrix degradation. Fast stress relaxation enhances cell spreading, proliferation and osteogenic differentiation of stem cell through integrin-RGD binding and clustering (Figure 1B). Stress relaxation has also been demonstrated to have the capability to alter chondrocyte phenotype and matrix deposition via modulating cell volume expansion (Lee et al., 2017). Slow relaxing environment restricts cell volume, leading to interleukin-1β secretion increase, which in turn up-regulates the genes related to matrix degradation and cell death. To fully mimic the mechanical and structural cues of native ECM, HA crosslinked with dynamic covalent bonds and fibrillar collagen type I are used to prepare IPN hydrogel with tunable stress relaxation (Lou et al., 2018). Cell spreading, fiber remodeling and focal adhesion formation are enhanced in the faster relaxation hydrogels.

### Self-healing

**Figure 2:**
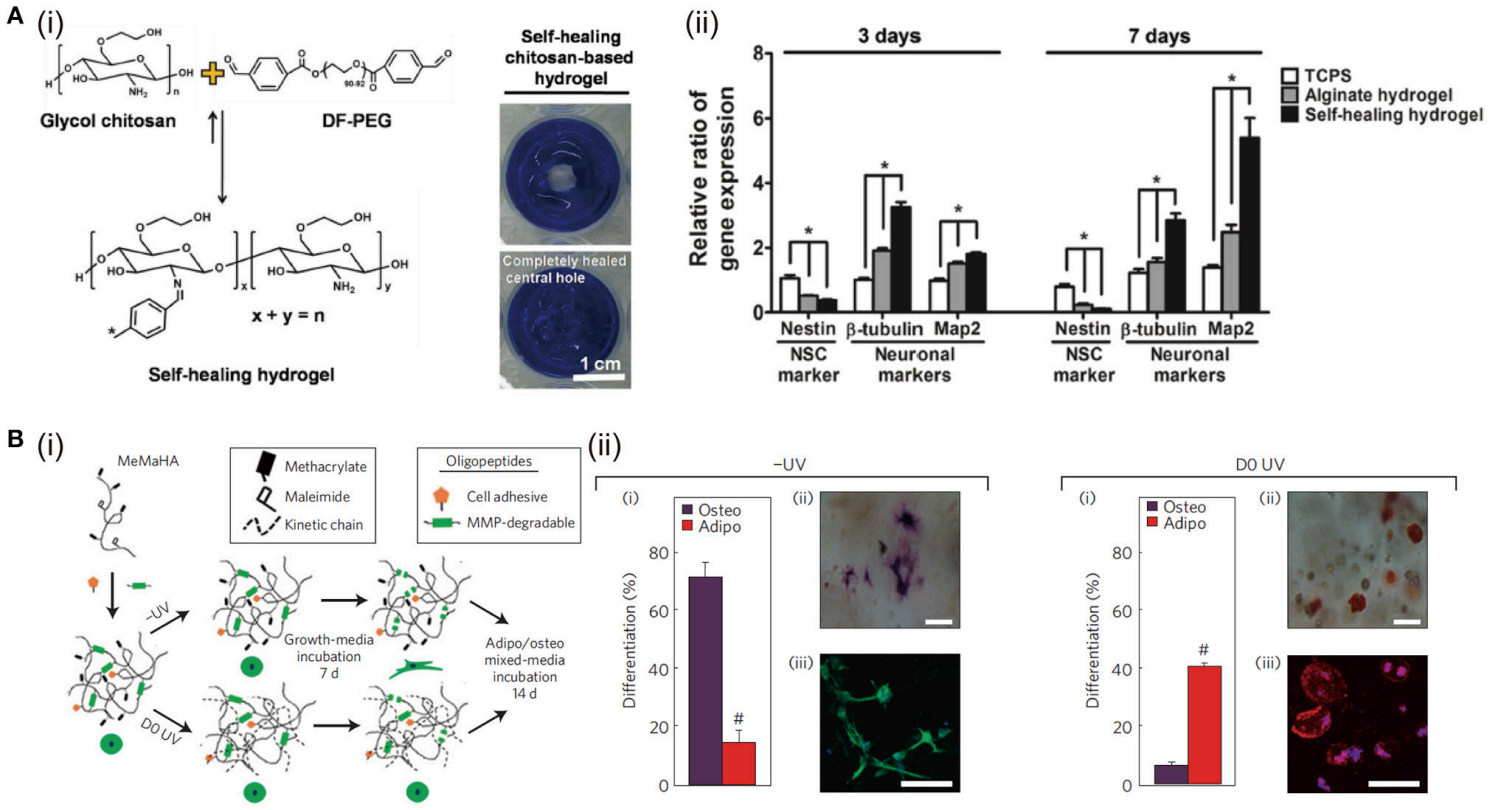
The influence of hydrogel self-healing and degradation on cell functions: **(A)** Self-healing hydrogel formed by crosslinking of benzaldehydes at both ends of difunctionalized PEG (DF-PEG) with glycol chitosan (i). The expressions of neuronal-related genes (nestin, β-tubulin, and Map2) of cells after 3 and 7 days of culture in 3D gels, *p < 0.05 (ii). Reproduced with the permission from Tseng et al. (2015), Copyright © 2013, John Wiley and Sons. **(B)** (i) Schematic of sequential crosslinking of MeMaHA using a primary addition and secondary radical polymerization to create -UV and D0 UV hydrogels. (ii) Percentage of hMSCs toward osteogenic or adipogenic differentiation in -UV or D0 UV hydrogels (#*p* < 0:005, *t*-test). Reproduced with the permission from Khetan et al. (2013), Copyright © 2013, Springer Nature.

Self-healing hydrogel is able to intrinsically and automatically heal the breaks, making itself back to original shape and mechanical property. This property is directly based on the reversible or dynamic crosslinking chemistry (Taylor and in het Panhuis, 2016). One of the representative self-healing hydrogels is prepared by glycol chitosan and benzaldehyde functionalized PEG via Schiff-base reaction (Tseng et al., 2015). The proliferation rate and differentiation tendency of neurosphere-like progenitors cultured in this self-healing hydrogel are enhanced (Figure 2A). Thrombin crosslinked fibrinogen can build a 3D network structure, which is used to prepare IPN structure after mixing with the above-mentioned chitosan-PEG hydrogel (Hsieh et al., 2017). This hydrogel encapsulated with vascular endothelial cells shows excellent self-healing and capillary-inducing capacity. Injection of this hydrogel promotes angiogenesis in zebrafish and mice. Dynamic acylhydrazone bond and Diels-Alder click covalent crosslinking are combined to prepare self-healing hydrogels with desirable mechanical property (Lü et al., 2017). These hydrogels increase the viability, reduces apoptosis of MSCs, and enhances bone regeneration in cranial bone defects. Guest-host crosslinking has also been used to prepare self-healing hydrogels. Cyclodextrin conjugated in one polymer backbone can work as the host to engulf hydrophobic moieties of another polymer chain to achieve self-healing. This self-healing property can further be selectively improved when the stimuli-responsive moiety, such as polyacrylamide-ferrocene with a reversible hydrophobic-charge transition at reduced/oxidized state, is included in the above hydrogels (Nakahata et al., 2011).

### Degradation

Covalently crosslinked hydrogels can undergo degradation through ester hydrolysis, enzymatic hydrolysis or photolytic cleavage of the polymer chains (Kharkar et al., 2013). Based on these mechanisms, hydrogels could be designed with good biodegradability and desirable degradation rate, working as temporary supports and being degraded and replaced by the regenerating tissues gradually (Bryant et al., 2004). For example, the photocrosslinkable PVA/PEG hybrid hydrogels with controlled degradation profiles have been used for cartilage TE. The degradation time can be tuned from less than 1 to 34 days by altering the ratio of PVA to PEG. The results show that the DNA and GAG contents increase with culture time and the neocartilaginous tissue at 6 weeks is homogeneously distributed in the PVA/PEG hydrogel with the ratio of 1:3, indicating the importance of degradation for TE (Martens et al., 2003). When implanted *in vivo*, HA-based hydrogel often shows too fast degradation to meet the requirement for cartilage tissue repair. To solve this limitation, sulfate groups are conjugated to HA to decelerate the hyaluronidase degradation rate. Sulfated HA displays slow degradation and enhances protein binding ability, promoting chondrogenesis of hMSCs with reduced hypertrophy (Feng et al., 2017).

Degradation of the hydrogel is a chemical process, but it can work as a dynamically physical stimulus to affect cell behaviors and functions for cell-matrix interaction, such as cell spreading, migration, proliferation, and differentiation, to further mimic the native ECM and improve the tissue regeneration. For example, photo-responsive hydrogels can be prepared from PEG derivatives containing nitrobenzyl ether photolabile moieties. When human mesenchymal stem cells (hMSCs) are cultured in the photodegradable hydrogel, cell spreading is enhanced after light exposure (Kloxin et al., 2010). Degradation of hydrogel is always accompanied with decrease of stiffness, making it complicated to discriminate the influences of degradation and stiffness. To decouple the influence from mechanical property of degradable hydrogels, hydrogels are designed to degrade while their mechanical properties remain unchanged. For example, HA functionalized with both maleimide and methacrylate is used to prepare hydrogels by thiol-ene crosslinking via MMP degradable crosslinker and photo-initiated crosslinking of the methacrylate. The MMP-cleavable peptide crosslinker permits cell-mediated degradation. The photo-initiated crosslinking can dynamically control the secondary crosslinking at the very beginning or after a period of cultivation. The stiffness of hydrogels with or without UV exposure is controlled at the same level by changing the molecular weight of HA macromers. When hMSCs are cultured in the tuned hydrogels, they exhibit different osteogenic and adipogenic differentiation. The results indicate that hMSCs differentiation is directed by degradation-mediated cellular traction, independent of the matrix mechanics. (Khetan et al., 2013) (Figure 2B).

## Control of chemical properties

The composition of hydrogels can highly affect the cell behaviors including viability, adhesion, spreading, proliferation, and differentiation (Ruoslahti and Pierschbacher, 1987). Hydrogel composition can be controlled by choosing different precursors and preparation methods. The methods used to tune chemical properties of functional hydrogels are summarized in Table 3.

**Table 3:** Control of chemical properties of hydrogels for TE applications.

**Original hydrogels**	**Approaches**	**Applications and Performance**	**References**
HA, alginate, chitosan, and PEG	Modifying hydrogel precursors with RGD peptides	Promoted cell adhesion and viability, enhanced cell proliferation and differentiation	Lee et al., 2015; Kim et al., 2016; Long et al., 2017; Sun et al., 2017
Polyacrylamide	Hybridization with GelMA	Improved biocompatibility of synthetic hydrogels	Han et al., 2017
PEG	Hybridization with HA	Increased chondrocyte number and sGAG and collagen production	Skaalure et al., 2014; Fu et al., 2017
PEG	Covalently tethered transforming growth factor-beta 1 (TGF-β1) to PEG hydrogel through thiol-ene reaction	Increased chondrogenic matrix deposition by immobilization of TGF-β1	Sridhar et al., 2014; Mao et al., 2017
GelMA	Hybridization with nanosilicates	Promoted osteogenic differentiation of preosteoblasts in a growth-factor-free microenvironment	Xavier et al., 2015
GelMA Collagen/Alginate	Hybridization with multiwalled CNTs Gold nanorod	Improved cell adhesion and maturation; enhanced cardiac tissue regeneration, exhibiting strong spontaneous and stimulated synchronous beating	Shin et al., 2013; Navaei et al., 2016a; Izadifar et al., 2018
Polyacrylamide	Hybridization with graphene oxide	Enhanced proliferation and myogenic differentiation of C2C12 cells, and combining electrical stimulation further enhanced myogenic gene expression	Jo et al., 2017

**Figure 3:**
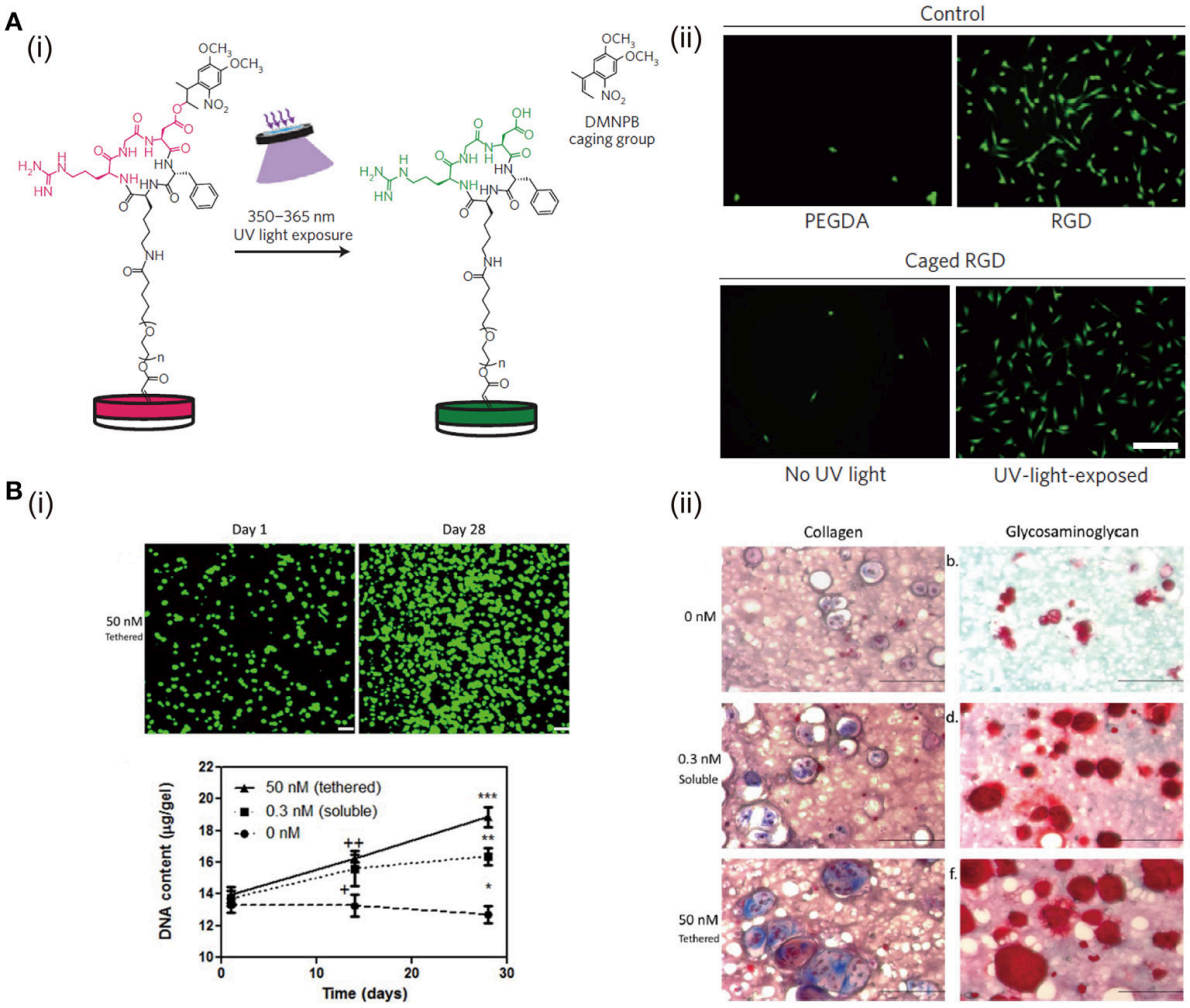
The effects of RGD peptides and growth factors in hydrogels on cell functions: **(A)** Schematic representation of the activation of caged RGD peptides in PEGDA hydrogels under light exposure (i). Cell adhesion and spreading can be enhanced after culture in hydrogels with RGD peptides and UV-light-exposed caged RGD peptides (ii). Reproduced with the permission from Lee et al. (2015), Copyright © 2015, Springer Nature. **(B)** ECM production of chondrocytes is enhanced after culture in PEG hydrogels with covalently tethered TGF-β. DNA content of chondrocytes exposed to 50 nM tethered TGF-β is the highest (i). Chondrogenic matrix (collagen and glycosaminoglycan) deposition is enhanced when exposing to TGF-β. The matrix produced in 50 nM (tethered) group is higher than that in 0.3 nM (soluble) group (ii). Reproduced with the permission from Sridhar et al. (2014), Copyright © 2014, John Wiley and Sons.

Cell adhesion ligand is a crucial biochemical component in ECM, because many types of cells have to adhere to their microenvironment through cell surface integrin to perform the relative functions and maintain their viability (Huettner et al., 2018). Naturally derived proteins, like collagen and gelatin, retain many cell adhesion ligands. However, polysaccharide-based natural materials and synthetic polymers are lack of these ligands. Hence, bioactive peptide modification has been used to improve the biochemical property of non-adhesive hydrogels. RGD peptide, as one of the potent adhesion ligands, plays an important role on cell adhesion and other functions. To study the impact of dynamic presentation of RGD in matrix on cell functions, a light-responsive PEGDA hydrogel is synthesized, in which a protecting group is used to temporally and spatially control the RGD presentation by transdermal light exposure (Figure 3A). PEGDA hydrogels with RGD peptides and light-induced uncaged RGD peptides support high number of adherent cells. On the other hand, PEGDA hydrogels without RGD modifications or with caged RGD support few adherent cells and the cells have round morphology. Besides, it has been reported that RGD peptides can be introduced to polymers, such as HA (Lee et al., 2015), alginate (Sun et al., 2017), chitosan (Kim et al., 2016), and PEG (Long et al., 2017), by chemical binding to fabricated bioactive hydrogels. These functionalized hydrogels show improved biological properties to promote cell adhesion, spreading, proliferation, and differentiation. For instance, RGD peptides promote the survival of MSCs in PEG hydrogels and induce the chondrogenic differentiation (Salinas and Anseth, 2008). RGD ligands in hydrogels increase the chondrogenic gene expression when the hydrogel matrices are loaded with dynamic mechanical force (Steinmetz and Bryant, 2011). Moreover, RGD density affects the redifferentiation of chondrocytes (Schuh et al., 2012).

Hybridization with natural polymers is another facile way to tune the biochemical property of hydrogels, which can change the composition of hydrogels in favor of promotion of cell function and TE. For example, GelMA is used to hybridize with PAM to prepare a photopolymerized hydrogel for cartilage tissue regeneration. Cells cultured in the hybrid hydrogel exhibit higher viability and proliferation rate than those cultured in pure PAM hydrogel (Han et al., 2017). What's more, HA/PEG hybrid hydrogel prepared via the SPAAC crosslinking shows excellent biocompatibility (Fu et al., 2017), which may be due to the fact that HA can bond to some receptors on cell membrane to affect both chondrocyte survival pathway and apoptotic pathway (Knudson and Knudson, 2004).

Besides hybridization with natural polymers or modification with biological moieties to improve the biochemical properties of hydrogels for TE, loading of growth factors and nanoparticles have been frequently adapted to improve hydrogel functions. Growth factors play important roles in cell growth, cell function determination, tissue regeneration, and organ development (Parker et al., 2016; Yan et al., 2018). Therefore, immobilization of growth factors in hydrogels will significantly improve the functionality of hydrogels. There are two strategies for immobilizing growth factors in hydrogels, namely physical, and chemical immobilization (Nguyen and Alsberg, 2014). Heparin can bind many types of growth factors through the strong electrostatic interactions. The immobilization of fibroblast growth factor-2 and vascular endothelial growth factor in heparin-modified PEG hydrogels has been shown to boost angiogenesis (Zieris et al., 2010). Compared with physical immobilization, chemical immobilization can further improve the stability, prolong the release of growth factors, and reduce the required amount. For example, transforming growth factor-beta 1 (TGF-β1) is covalently tethered to PEG hydrogel through thiol-ene reaction (Sridhar et al., 2014; Mao et al., 2017). Chondrocytes cultured in PEG hydrogel immobilized with TGF-β1 show higher DNA content and chondrogenic matrix production than the cells cultured in PEG hydrogel with soluble TGF-β1 (Figure 3B).

**Figure 4:**
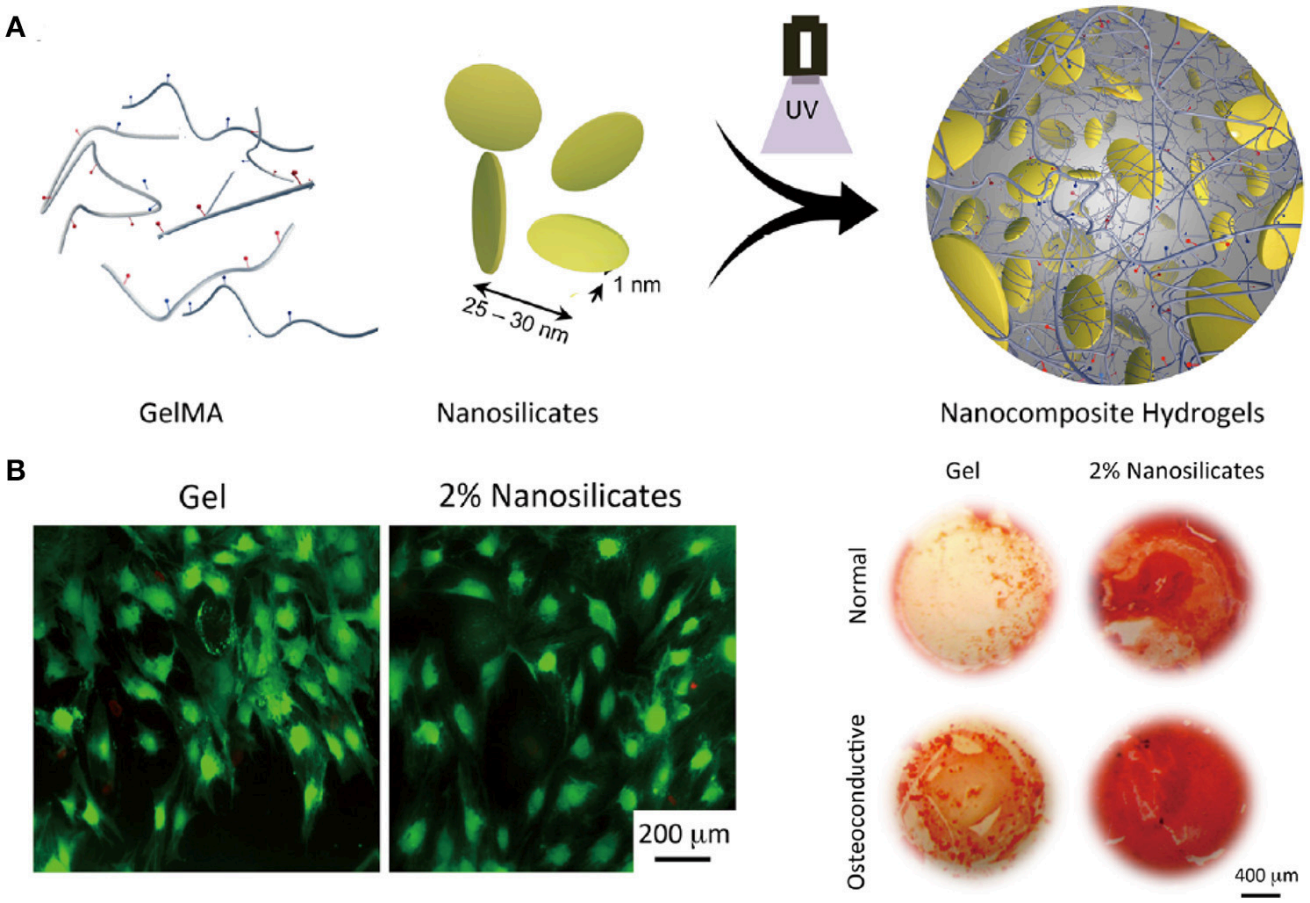
Schematic illustration of fabrication of nanocomposite hydrogels from GelMA and nanosilicates by photocrosslinking **(A)**. Nanosilicate-loaded gelatin hydrogels can support cell adhesion and spreading (live/dead staining) and enhance inorganic calcium deposition in normal and osteoconductive media (Alizarin Red S staining) **(B)**. Reproduced with the permission from Xavier et al. (2015), Copyright © 2015, American Chemical Society.

A variety of nanoparticles have also been incorporated into different kinds of natural or synthetic polymer networks to prepare nanocomposite hydrogels for TE. The incorporation of nanoparticles can provide not only higher mechanical properties, but also the possibility to tune biochemical characteristics of the 3D network. For examples, nanosilicates are incorporated into GelMA hydrogels to obtain a bioactive nanocomposite (Xavier et al., 2015). Not only the mechanical strength of the hydrogel is enhanced, osteogenic differentiation of preosteoblasts is also promoted (Figure 4). In another study, nanosized hydroxyapatite is incorporated into a PEG hydrogel aiming to produce highly tough matrix for bone TE. After incorporation, the morphologies of these hydrogels show highly interconnected porous structures. What's more, the presence of hydroxyapatite nanoparticles can provide osteoblast cells adhesion and bioactive attachment sites compared with pure PEG hydrogels (Gaharwar et al., 2011). For cardiac tissue regeneration, conductive nanoparticles like carbon nanotubes are added into GelMA hydrogels, resulting in improved cell adhesion and maturation (Shin et al., 2013). The percentage of cell retention and viability on carbon nanotubes nanocomposite GelMA hydrogel is higher than those on pristine GelMA. The mature cell/hydrogel sheet with very good electrophysiological and mechanical properties exhibits strong spontaneous and stimulated synchronous beating.

## Structural control of hydrogels

Microstructure is another critical factor to affect cell activities and functions because the optimized architecture of engineered tissue has the function to organize multiple cell types for TE (Stevens et al., 2013). Hydrogels with microarchitectures are briefly classified into microporous, channel-bearing, double-ring, multilayered, and hierarchically structured ones. The structure types of functional hydrogels and their influence on cell functions and tissue regeneration are summarized in Table 4.

**Table 4:** Structural control of hydrogels and TE applications.

**Structural hydrogels**	**Materials**	**Approaches**	**Applications and Performance**	**References**
Porous structure	Alginate HA	Mixing with gelatin particles prepared by water/oil emulsion	Chondrogenic matrix secretion and gene expression were improved in alginate and HA porous hydrogels.	Fan and Wang, 2015; Leong et al., 2016
	GelMA	Mixing with gelatin cubes prepared by mesh-cutting	Chondrocytes migration and proliferation were enhanced in porous structures.	Li et al., 2017a
	GelMA	Gelatin, alginate, and HA porogens prepared by water/oil emulsion can be degraded to specific stimuli including temperature, chelating and enzymatic digestion, respectively.	Increased cell proliferation and spreading, and enhanced type II and X collagen production happened in the hydrogel with dynamic pore formation	Han et al., 2013
Channel structure	2-hydroxyethyl methacrylate, agarose or GelMA	Embedding PVA sacrificial templates	High cell viability in bulk hydrogel was achieved by this channel structures.	Tocchio et al., 2015
	Gelatin	Embedding and dissolving solvent-spun PNIPAM microfibers		Lee et al., 2016
	Agarose/alginate/PEG/Fibrin/Matrigel	Carbohydrate-glass fibers were 3D printed and removed after surround hydrogel formation.	Good biocompatibility and enhanced nutrition diffusion.	Miller et al., 2012
Double-ring structure	GelMA and hydroxyapatite	Osteon-like concentric double-ring structure was prepared via photolithography and self-assembly.	HUVECs and MG63s were encapsulated in the inner and outer ring, working as blood vessel tubule and bone, respectively.	Zuo et al., 2015; Wei et al., 2017
Bilayered structure	Transglutaminase factor XIII crosslinked PEG hydrogels	Chondrocytes and MSCs were encapsulated in different hydrogel layers functionalized with TGF-β3 or BMP-2.	Endochondral bone or stable cartilage can be developed at an ectopic site without the need of a predifferentiation process *in vitro*.	Stüdle et al., 2018
	Agarose	Chondrocytes and MSCs were encapsulated in the top and bottom layer agarose hydrogel for osteochondral TE.	Coculture of chondrocytes and MSCs in different environment showed potential for osteochondral TE.	Sheehy et al., 2013
	PEG-derivative	Top layer with low RGD concentration and soft stiffness was designed for chondrogenesis of MSC; bottom layer was prepared with high RGD concentration and high stiffness for osteogenesis of MSC.	Spatial presentation of physiochemical cues combined with dynamic mechanical stimulation could regulate the differentiation of MSCs.	Steinmetz et al., 2015
Hierarchical structure	PEG-derivative	Hierarchical vessels were fabricated by multiphoton lithography.	Human bone marrow-derived hS5 stromal cells exhibited high viability for a long culture period.	Arakawa et al., 2017
	POMaC and collagen	POMaC made scaffold with nanopores and micro-holes was prepared by using 3D stamping technique.	The incorporation of nanopores and micro-holes enhanced permeability, and permits intercellular crosstalk and extravasation.	Zhang et al., 2016

### Microporous hydrogels

**Figure 5:**
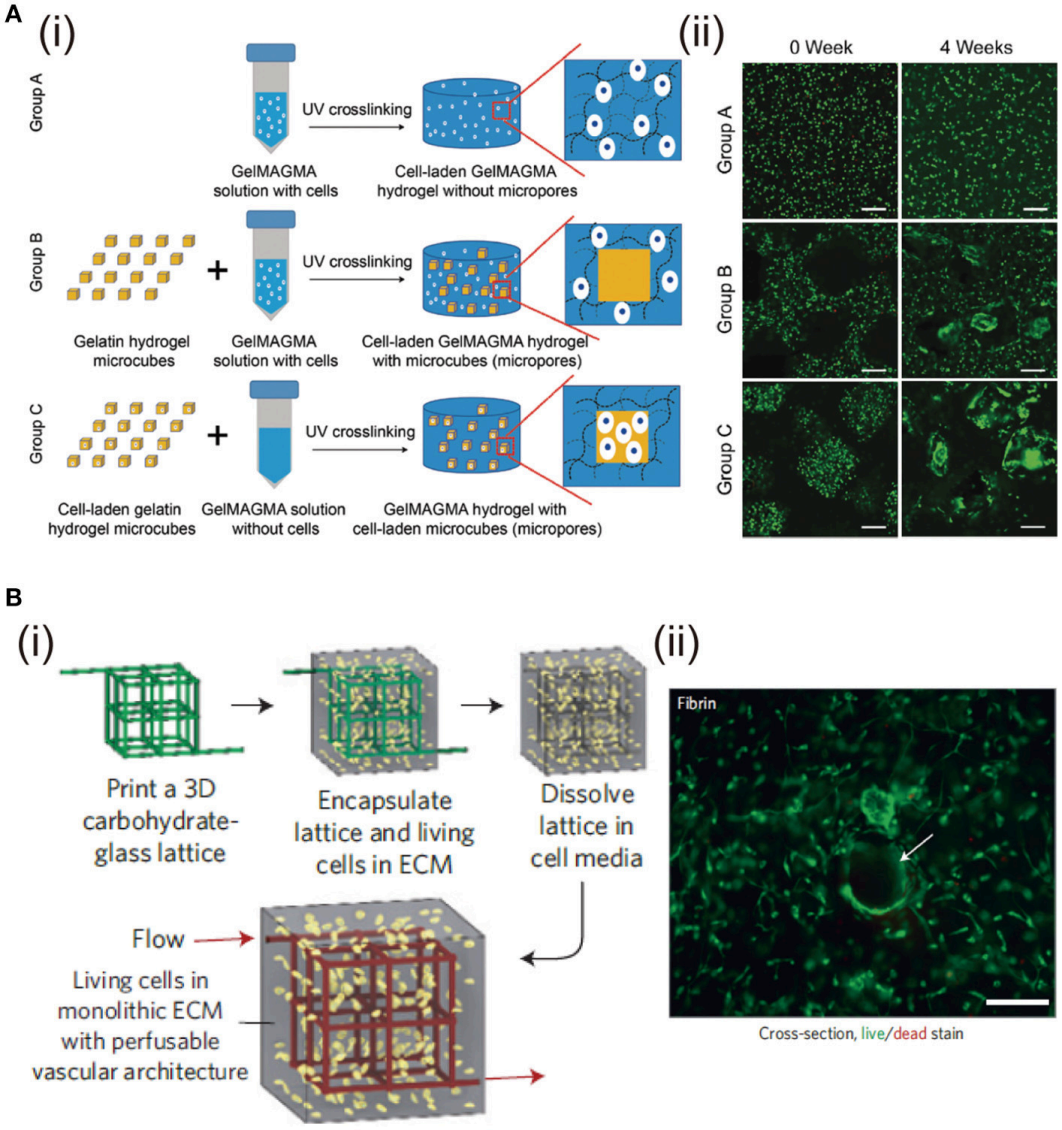
Fabrication of porous and channel hydrogels and the effects on cell functions. **(A)** Preparation scheme of GelMAGMA hydrogels with or without microporous structures. (i). Live/dead staining of chondrocytes in the hydrogels after UV crosslinking (0 week) and after 28 days of *in vitro* culture (4 weeks) (ii). Scale bar: 200 mm. Reproduced with the permission from Li et al. (2017a), Copyright © 2017, Royal Society of Chemistry. **(B)** Fabrication of hydrogel with channel structure by dissolving 3D printed carbohydrate-glass lattice (i). Representative live/dead image of HUVEC and 10T1/2 co-cultured in the interstitial space of a fibrin gel (ii). Cells survive and spread near open channels (highlighted with white arrow). Scale bar: 200 μm. Reproduced with the permission from Miller et al. (2012) Copyright © 2012, Springer Nature.

The structure of bulk hydrogels is dense polymers with absorbed water and nano-sized pores within the network (Hoffman, 2012), in which, the nano-sized pores are too small to promote cell migration, proliferation and ECM diffusion. Thereby, microporous structures have been proposed. The effects of different pore structures and pore size ranges have been studied for cell culture and TE (Zhang et al., 2014; Chen et al., 2016). Various technologies including solvent casting, particle leaching, freeze-drying, and gas foaming can be used to adjust hydrogel porosity (Annabi et al., 2010). Among them, stimuli-responsive porogens, such as gelatin, alginate and HA, are used to create cell-laden hydrogels with tunable porosity. These porogens can be removed by specific stimuli including temperature, chelating, and enzymatic digestion, respectively (Han et al., 2013). Similarly, gelatin microspheres fabricated by water/oil emulsion are used to generate micropores in alginate (Leong et al., 2016) and HA (Fan and Wang, 2015) hydrogels. Plenty of micropores are left after gelatin microbeads are dissolved at 37°C. Chondrocytes cultured in the porous hydrogels show high proliferation and ECM secretion. Gelatin microparticles have also been used to prepare microporous hydrogels for bone TE (Vo et al., 2016). The pore-forming gelatin microparticles can be used not only for generation of micropores, but also for introduction of living cells in the micropores. Cell-laden gelatin microcubes are prepared by a mesh-cutting method and used to prepare microporous hydrogels with promotive capacity of cell proliferation (Li et al., 2017a). Chondrocytes cultured in the microporous hydrogels show high proliferation and cells prefer to migrate into the microporous cavities (Figure 5A).

### Channel-bearing hydrogels

Cells must reside with 100–200 μm from adjacent capillary blood vessels to remain viable or else they will undergo necrosis due to insufficient oxygenation and nutrition diffusion (Carmeliet and Jain, 2000). Therefore, hydrogels with micro-channels are highly needed to vascularize artificial tissue or study cell behaviors in a vascular structure, in particular for the regeneration of large and complex tissues and organs. Multiple strategies and techniques have been developed for preparation of channel-bearing hydrogels for TE applications.

PVA-based sacrificial templates are fabricated into branched fluidic architectures by casting (Tocchio et al., 2015). The architecture obtained after washing exhibits promoted regeneration of hierarchically branched endothelium and high cell viability inside bulk hydrogels formed by 2-hydroxyethyl methacrylate, agarose or GelMA. To prepare a complex capillary-like micro-channel vascular structure, solvent-spun PNIPAM microfibers are fabricated and used as temperature-responsive templates (Lee et al., 2016). The PNIPAM microfibers are spun and maintained at a temperature above 32°C. Cell-suspended gelatin precursor solution is kept at 37°C prior to embedding the templates. After complete gelation of the gelatin hydrogel, the PNIPAM microfibers are removed by immersion in cell culture medium at room temperature to generate the capillary-like micro-channel vascular structure. Cell viability is higher than 96% during 7 days of cultivation in the perfused micro-channel hydrogel. Carbohydrate-glass microfibers are printed to induce vascular architecture in bulk hydrogels, which can be removed by cell medium perfusion (Miller et al., 2012). The micro-channel hydrogels demonstrate good biocompatibility and enhanced nutrition diffusion. Combination of carbohydrate glass material with 3D printing provides independent control of vascular network geometry, endothelialization, and extravascular tissue formation (Figure 5B).

### Double-ring structural hydrogels

**Figure 6:**
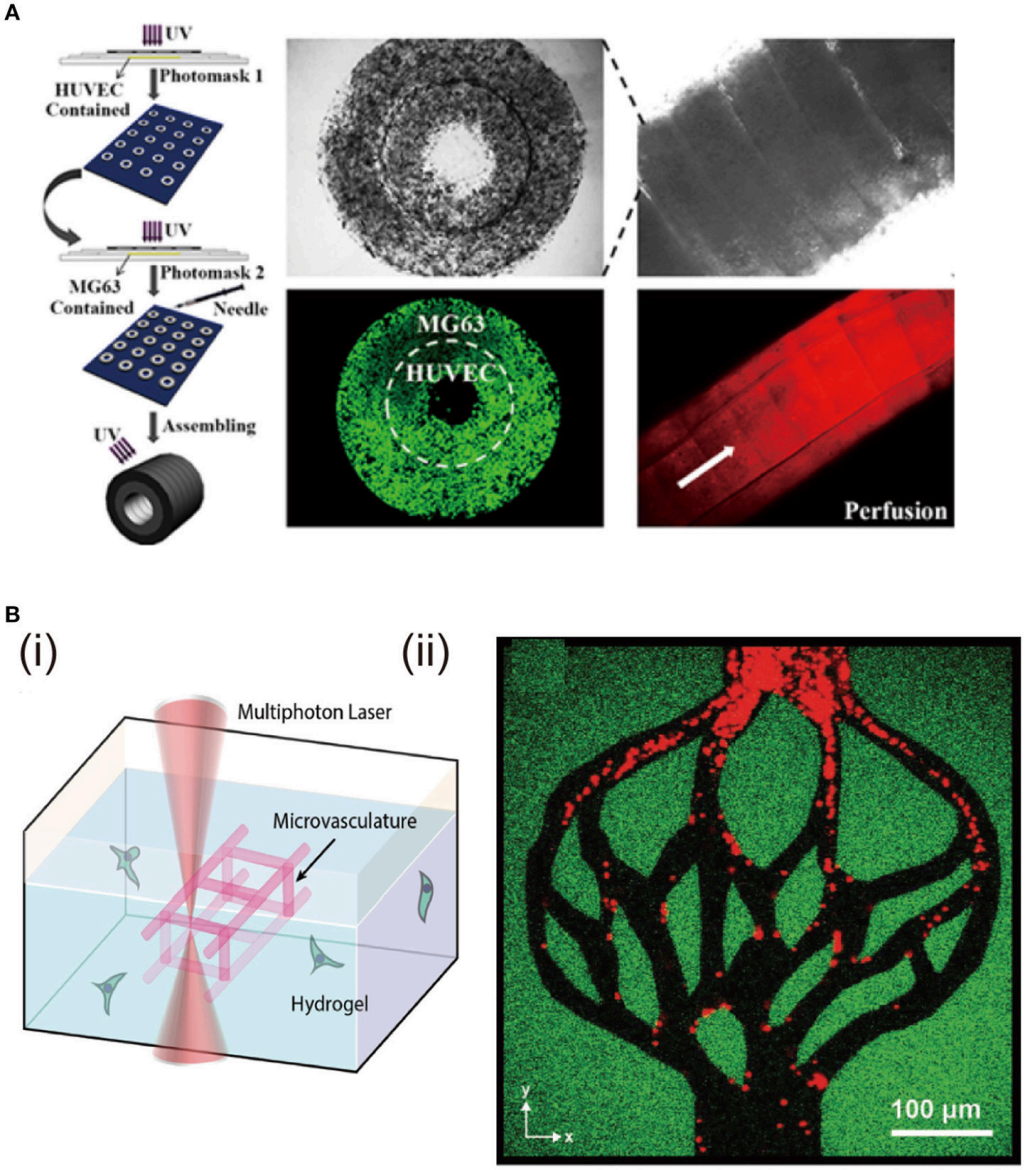
Fabrication of double-ring and hierarchically structural hydrogels and the effects on cell functions. **(A)** Fabrication of hydrogels with osteon-like double-ring structure by photolithograph and self-assembly. Cell viability of MG63s and HUVECs encapsulated in the outer ring and inner ring of the osteon-like module. Reproduced with the permission from Zuo et al. (2015), Copyright © 2015, American Chemical Society. **(B)** Degradation of the hydrogel through oNB photocleavage (i). Creation of hydrogels with hierarchical vascular structure by programmable photodegradation (ii). Reproduced with the permission from Arakawa et al. (2017), Copyright © 2017, John Wiley and Sons.

Osteon-like concentric double-ring structure is prepared with hybrid hydrogels of GelMA and hydroxyapatite via photolithography technology and self-assembly (Zuo et al., 2015). Human umbilical endothelial cells (HUVECs) are encapsulated in the inner ring to mimic blood vessel tubules, while human osteoblast-like cells (MG63s) are located in the outer ring as bone part (Figure 6A). Expression of angiogenesis-related and osteogenesis-related genes is promoted. Osteon-like fibers with HUVECs and MG63s separately encapsulated are constructed with the combination of photolithography and microfluidic chip techniques (Wei et al., 2017). RGD modified alginate precursor with photoreactive functionalization is used for the hydrogel preparation because the modified alginate possesses cell adhesive sites and UV/Ca^2+^ crosslinkable properties. This not only satisfies cell proliferation but enhances the relative gene expression and protein secretion in the osteon-like architecture.

### Multilayered hydrogels

ECM composition and structure of cartilage and bone tissues have an obvious difference. The biochemical and biophysical microenvironments surrounding the cells in cartilage and bone are also different. To regenerate full-thickness articular cartilage defects, bilayered hydrogel architecture is designed. For example, bilayered agarose hydrogels are prepared for osteochondral tissue repair (Sheehy et al., 2013). The top hydrogel layer is encapsulated with chondrocytes, while the bottom layer is seeded with MSCs. After 49 days of *in vitro* cultivation, the top layer promotes production of cartilaginous ECM, while the hypertrophy of MSCs encapsulated in the bottom layer is decreased. The results indicate the bilayered structure has a great potential for spatially separated coculture of chondrocyte and MSCs and osteochondral tissue regeneration. Bilayered structure can also provide tunable biochemical and mechanical microenvironment for differentiation of MSCs. For example, the top hydrogel layer fabricated with low RGD concentration and soft matrix can promote chondrogenesis of MSCs, while, the bottom layer designed to have high RGD amount and stiff mechanical property can promote osteogenesis of MSCs (Steinmetz et al., 2015).

### Hierarchically structured hydrogels

Hierarchical architecture exists in living tissues. For example, blood vessels possess many scales of size to adapt to tissue requirements. They include large arteries, smaller arterioles and smallest capillaries, which have the functions to permit large blood volume flow and support mass transport via diffusion (Michiels, 2003). The above mentioned methodologies for channel structures have limitations to introduce such hierarchical structures in cell-laden hydrogel constructs, especially for the size of diameter less than 150 μm (Lee et al., 2014). Multiphoton lithography-assisted photoscission, as a cytocompatible fabrication strategy, is used to create hierarchical vessels spanning nearly all the size ranges in the human body (Arakawa et al., 2017) (Figure 6B). The hydrogel network is crosslinked by azide-alkyne cycloaddition between PEG tetrabicyclononyne and diazide-functionalized peptide. The photo degradation is based on the ortho-nitrobenzyl ester (oNB) moiety that undergoes photolysis upon exposure to pulsed near-infrared light (DeForest and Tirrell, 2015). Human bone marrow-derived hS5 stromal cells are encapsulated inside the hydrogel, followed by preparation of photodegradable vessels. The hS5 cells are viable during the whole culture period. This complex and hierarchical structures can maintain cell viability and functions, which is essential for regeneration of heterogeneous tissues. Furthermore, the hierarchical vascular structure is promising for investigation of endothelialization and blood-capillary interaction in the various environments.

3D stamping technique can also be used to prepare hydrogels with hierarchical structures. AngioChip with hierarchical vascular and porous architecture is prepared by this method (Zhang et al., 2016). Nanopores and micro-holes are fabricated into the vascular walls to promote molecular exchange and cell migration. The permeability for large molecular (70 kDa TRITC-dextran) of AngioChip with micro-holes is more than 4 times higher than that of the counterparts without micro-holes. A confluent endothelium is exhibited on the microchannel surface and endothelial sprouts are observed through the micro-holes. Human embryonic stem cell-derived hepatocytes and hMSCs are encapsulated in a collagen matrix and seeded into the parenchymal space to engineer the AngioChip. Urea secretion per cell from this hierarchical construct is much higher than that of collagen sandwich control. Furthermore, this complex hierarchical structural AngioChip is also used for *in vitro* regeneration of cardiac tissue models and implantation for direct surgical anastomosis. Due to the complex and hierarchical architectures, cell viabilities and relative functions are enhanced.

## Conclusion and outlooks

Control of the structures and physiochemical properties of hydrogels is important for their utilization in TE. Cells first sense the signals from surrounding hydrogel matrix, which makes the physiochemical properties of hydrogel critical for cell functions. Various structures of hydrogels can further alter the microenvironments and provide the possibility of flow stimulus by micro-channel structure or co-culture microenvironment by spatially layered structures. A variety of preparation methods and functional hydrogels have been developed for TE of various tissues and organs. Despite of these progresses, hydrogels with further improved biochemical and biophysical properties, as well as biomimetic structures are deserved to be further studied. The explored hydrogels until now have some similar but not completely matched functions to the *in vivo* cell-surrounding microenvironments. Dynamically responsive biophysical and biochemical stimuli and spatiotemporally controlled architecture may become the main direction of design and preparation of functional hydrogels to realize the complete functionality for TE applications.

## Author contributions

All authors listed have made a substantial, direct and intellectual contribution to the work, and approved it for publication.

### Conflict of interest statement

The authors declare that the research was conducted in the absence of any commercial or financial relationships that could be construed as a potential conflict of interest.
